# Present value of the Urethral mobility test as a tool to assess Stress urinary incontinence due to Intrinsic sphincteric deficiency

**DOI:** 10.1038/s41598-020-77493-1

**Published:** 2020-12-02

**Authors:** Daniela Robledo, Laura Zuluaga, Alejandra Bravo-Balado, Cristina Domínguez, Carlos Gustavo Trujillo, Juan Ignacio Caicedo, Martín Rondón, Julián Azuero, Mauricio Plata

**Affiliations:** 1 Department of Urology, Hospital Universitario Fundación Santa Fe de Bogotá and Universidad de Los Andes School of Medicine, Carrera 7 No. 118-09, Unidad Renal, piso 3, 110111 Bogotá, D.C. Colombia; 2 Department of Clinical Epidemiology and Biostatistics, Pontificia Universidad Javeriana, Bogotá, D.C. Colombia

**Keywords:** Urology, Urinary incontinence

## Abstract

Q-tip test offers a simple approach for identifying urethral hypermobility. Considering surgical treatment, stress urinary incontinence (SUI) must be classified and the contribution of intrinsic sphincter deficiency (ISD) and/or urethral hypermobility must be determine. We believe there's a correlation between abdominal leak point pressure (ALPP) and urethral mobility degree, and the aim of this study is to explore it using Q-tip. We conducted a prospective study, between years 2014 and 2016. Females over 18 years presenting with signs and symptoms of SUI according to the 2002 ICS Standardization of Terminology were included. Assessment was made with the International Consultation on Incontinence Questionnaire Short Form (ICIQ-SF), the Q-tip test and invasive urodynamics. Urethral mobility (UM) and ALPP were analyzed. We built two composite variables based on reported risk factors for ISD, defined as composite variable A (equal to a Q-tip test < 30° AND ICIQ-SF ≥ 10 points) and composite variable B (equal to low urethral mobility AND/OR hypoestrogenism AND/OR history of radiotherapy AND/OR previous pelvic surgery). Correlation analyzes were made according to the type of variable. A total of 221 patients were included. Incontinence was rated as moderate and severe by 65.3% and 6.8%, respectively. The analysis showed a 61.75%, 51.61% and 70.6% agreement between ALPP and UM, ALPP and composite variable A and ALPP and composite variable B respectively. Correlation and concordances were low (r = 0.155, r_s = − 0.053 and r_s = − 0.008), (rho_c = 0.036, k = 0.116 and k = 0.016). Neither the degree of UM, nor the composite variables, correlate or agree with urethral function tests in UDS, suggesting that the ALPP cannot be predicted using the Q-tip test or the ICIQ-SF for classifying patients with SUI.

## Introduction

Female urinary incontinence (UI) is a prevalent condition with substantial impact in patients’ daily activities and their quality of life, and therefore^1^, a frequent cause for consultation^2^. Several clinical assessment tools and criteria are available to classify UI to offer the ideal treatment accordingly^3^. For stress urinary incontinence (SUI) in particular, a distinction must be made to determine the contribution of intrinsic sphincter deficiency (ISD) and/or urethral/bladder neck hypermobility, considering this will guide the treatment and probably its surgical approach^4^.

UDS may be considered the most reliable test for achieving an objective preoperative evaluation of patients with SUI^5^, given that it allows to measure the leak point pressure (LPP), which in turn provides insight on the underlying mechanism^4,6^. However, even if theoretically the urodynamic study (UDS) optimizes the selection of cases and the choice of surgical procedures, to date there is no consensus on whether performing UDS results in better surgical outcomes^7,8^. Therefore, interest has been raised about the value of other tests to guide the diagnosis of ISD and/or to predict its surgical outcome.

The Q-tip test offers a simple, office-based approach for identifying urethral hypermobility. It is performed by introducing a cotton swab through the urethral meatus to the bladder neck, and measuring its displacement with a goniometer during Valsalva maneuver. Although is invasive and that several European guidelines (e.g. EAU, NICE/UK, French) either do not offer comment or recommend its use it is still considered in American guidelines such as the latest Surgical Treatment of Female Stress Urinary Incontinence (SUI): AUA/SUFU Guideline^9^ and we can see on recently published evidence that it is still part of the repertoire of available diagnostic and follow-up tests for stress urinary incontinence for certain study groups^10,11^. Q-tip ability in predicting UDS findings is still unclear for the above our aim is to assess the level of correlation and agreement between ALPP and the degree of urethral mobility measured by the Q-tip test in females with SUI.

## Materials and methods

Institutional review board of clinical studies and ethics committee approval was obtained (Protocol no. CCEI-2158-2013) at Fundación Santa Fe de Bogotá. The study was developed following good clinical practices and the declaration of Helsinki in compliance with ethical institutional and international standards.

Females with complaints if SUI, aged 18 years and older who attended our Incontinence Care Center between September 2014 and September 2016 were eligible. Patients had to qualify and consent for assessment UDS.

We adhere to the definitions of the 2002 International Continence Society (ICS) Standardization of Terminology of Lower Urinary Tract Function Report^1^ and the International Urogynecological Association (IUGA)/ICS Joint Report on the Terminology for Female Pelvic Floor Dysfunction published in 2010^12^ Our Incontinence Care Center also complies with the ICS’ good urodynamic practices published to the most recent date^13^.

Exclusion criteria were: to suffer from any neurological condition affecting the function of the urinary tract, history of urethral stricture, acute urethral inflammatory disease, active urinary tract infection, urodynamic evidence of detrusor overactivity, history of Onabotulinum Toxin A bladder neck injection in the last 6 months, and suffering from any active mental or psychiatric conditions that may hinder adequate completion of the medical consultation, UDS or questionnaires. A small group of patients presented symptoms corresponding to over active bladder but they are the resulting patients who have stress UI with some urge component.

Per protocol at our Incontinence Care Center, all patients undergo a previous medical history assessment by a urologist/urologist in training, with specific interest directed towards pelvic floor and incontinence risk factors such as number of pregnancies and deliveries, pelvic and genital surgery (specific, with technique and approach noted when the information was available), pelvic organ neoplasia and modality of treatment (e.g. surgery, radiotherapy, brachytherapy, combined etc.), smoking status and previous history, menopause and estrogen replacement therapy use. Infirmary personnel measure vital signs, weight and height. Body mass index is calculated to determine if the patient is overweight or obese. Then, a symptoms’ questionnaire (i.e. ICIQ-SF for females) is filled out. Informed consent is carried out separately for the UDS procedure and for participation in the study.

### The Q-tip test and urodynamic study

An uroflowmetry is performed as determined by the patient’s normal desire to void. Then, the patient is asked to place herself in the lithotomy position. Inspection of the genitals is performed by a urologist and findings such as prolapse (using the POP-Q classification system)^14^, perceived urethral hypermobility, perineal tear (findings on inspection of prior trauma during childbirth) and hypoestrogenism (defined as a qualitative dichotomous variable based on physical exam findings of loss of genital trophism usually related but not exclusive to menopause) are recorded on the clinical chart.

Cleaning and disinfection proceeds. We apply lidocaine jelly (2%) to lubricate the cotton-tipped swab and the meatus before insertion. The Q-tip is then inserted transurethrally calculating around 2–4 cm before resistance is met; this represents the urethrovesical junction. At this point, the protractor is located parallel to the excel portion of the Q-tip and Valsalva maneuver is elicited. The maximum urethral angle is measured 3 times and the average measure is recorded. Urethral hypermobility is defined as an angle of more than 30 degrees.

Cystometry and Pressure-Flow Study are performed according to the Best Urodynamic practices published to date^13,15^. We use the Aquarius TT system from Laborie medical technologies. Equipment is calibrated against atmospheric pressure, reference level being the superior edge of the symphysis pubis, with the patient seated, using a 6–7 Fr double-lumen vesical catheter and an 8 Fr rectal catheter with a latex balloon filled with 8 mL of distilled water. Then, transducers are connected and cough is elicited to verify adequate pressure measurement.

Stress test is performed in the absence of detrusor overactivity, at approximately half the maximum bladder capacity. Also, above 100 mL of instilled volume, patients are asked to cough every 100 mL and if leakage occurs, it is recorded. First sensation and urinary urgency are also recorded. The ALPP was recorded as the lowest value of intentionally increased intravesical pressure that provokes urinary leakage in the absence of a detrusor contraction, following the ICS^1^.

### Statistical analysis

We calculated a sample of 220 subjects to find a correlation and agreement of 0.85 and a standard deviation of 0.05. Patients were assessed using the ICIQ-SF, the Q-tip test and UDS. In addition to evaluating correlation and concordance between ALPP and the Q-tip test, we created and ran tests using two different composite variables. Composite variable A incorporated urethral mobility < 30° and ICIQ-SF ≥ 10 (low UM + ICIQ-SF), while composite variable B was composed of low UM hypoestrogenism, history of radiotherapy and previous pelvic surgery. Severity of UI, its subtypes and median duration of symptoms will be considered. Other variables related to obstetric history and pelvic organ prolapse were taken into account, Pelvic Organ Prolapse Quantification system (POP–Q) was used to define it; smoking history, pelvic floor surgery and menopause were also assessed.

A univariate analysis using frequencies for discrete variables and measures of central tendency and dispersion for continuous variables was performed using STATA statistical software version 14.0.

To evaluate association between continuous variables, Lin’s concordance correlation (rho_c), Pearson correlation (r), reduced major axis regression and Bland–Altman plots were used. For categorical variables, we used Spearman correlation (r_s) and Cohen’s kappa coefficient (k).

## Results

A total of 221 female patients were included, with a median age of 56 with an interquartile range (IQR) of 44.5–65 years. Incontinence was reported as mild, moderate and severe according to patient perception by 60 (27.8%), 141 (65.3%) and 15 (6.8%) patients, respectively. SIU was the main complain in 171 (79.2%) patients, followed by mixed UI (n = 36; 16.7%) and urge UI (n = 9; 4.2%). The median duration of symptoms was 24 (12–36) months and 82.6% had a positive cough stress test. Baseline characteristics and associated factors are summarized in Table 1. ALPP was categorized as > 90 cm H2O (n = 120, 55%), 61–90 cm H2O (n = 60, 27.5%) and ≤ 60 (n = 38, 17.5%) cm H2O. Mean ALPP was 99.4 ± 34.8 cm H2O. Regarding the Q-tip test, 91 (41.2%) women had fixed urethra (< 30°) while the rest showed a degree of urethral mobility ≥ 30°. Other clinical characteristics are summarized in Table 2. Median ICIQ-SF score was 14 (IQR 11–16) points and median QoL was 8 (IQR 11–16) points. Mean ALPP in urethral mobility < 30° was 113 (IQR 82–132) and urethral mobility ≥ 30° was 91 (IQR 70–120).

**Table 1:** Baseline characteristics and associated factors for UI.

	**n = 221**
BMI (n, %)*	
Normal	81 (37.3)
Overweight	117 (53.9)
Obese	19 (8.8)
History of pregnancy (n, %)	213 (96.4)
Number of pregnancies (median, IQR)	3 (2–4)
Number of vaginal deliveries (median, IQR)	2 (1–3)
Cigarette smoking (n, %)*	28 (12.8)
Menopause (n, %)*	131 (60.4)
Previous pelvic floor surgery (n, %)*	21 (9.9)
History of hysterectomy (n, %)*	70 (32.0)
Anterior POP (n, %)*	
No	70 (32.4)
Stage I	67 (31.0)
Stage II	71 (32.9)
Stage III	7 (3.2)
Stage IV	1 (0.5)
Posterior POP (n, %)*	
No	117 (54.2)
Stage I	63 (29.2)
Stage II	29 (13.4)
Stage III	6 (2.8)
Stage IV	1 (0.5)
Perineal tear (n, %)*	50 (23.8)

*BMI* body mass index; *POP* pelvic organ prolapse.

*Valid percentages due to missing data.

**Table 2:** Clinical variables.

	n = 221
Frequency of leakage (n, %)*	
Never	1 (0.5)
About once a week or less	14 (6.4)
2–3 times a week	40 (18.2)
Once a day	34 (15.5)
Several times a day	109 (49.5)
All the time	22 (10.0)
Amount of leakage (n, %)*	
None	2 (0.9)
Small	117 (53.2)
Moderate	80 (36.4)
Large	21 (9.5)
When does urine leak? (n, %)*	
Never	1 (0.5)
Before I can get to the toilet	42 (19.1)
When I cough or sneeze	152 (69.1)
When I am asleep	9 (4.1)
When I am physically active/exercising	167 (75.9)
When I have finished urinating and I am dressed	16 (7.3)
For no obvious reason	17 (7.7)
All the time	5 (2.3)

We found a 61.75%, 51.61% and 70.6% agreement between ALPP and urethral mobility, ALPP and the composite variable A and ALPP and the composite variable B, respectively. The concordances between these variables were very poor (rho_c = 0.036, k = 0.116 and) k = 0.016) for each one. It was also shown negative correlation between these variables (r = 0.155 and r_s = − 0.053, r_s = − 0.008) respectively.

**Figure 1:**
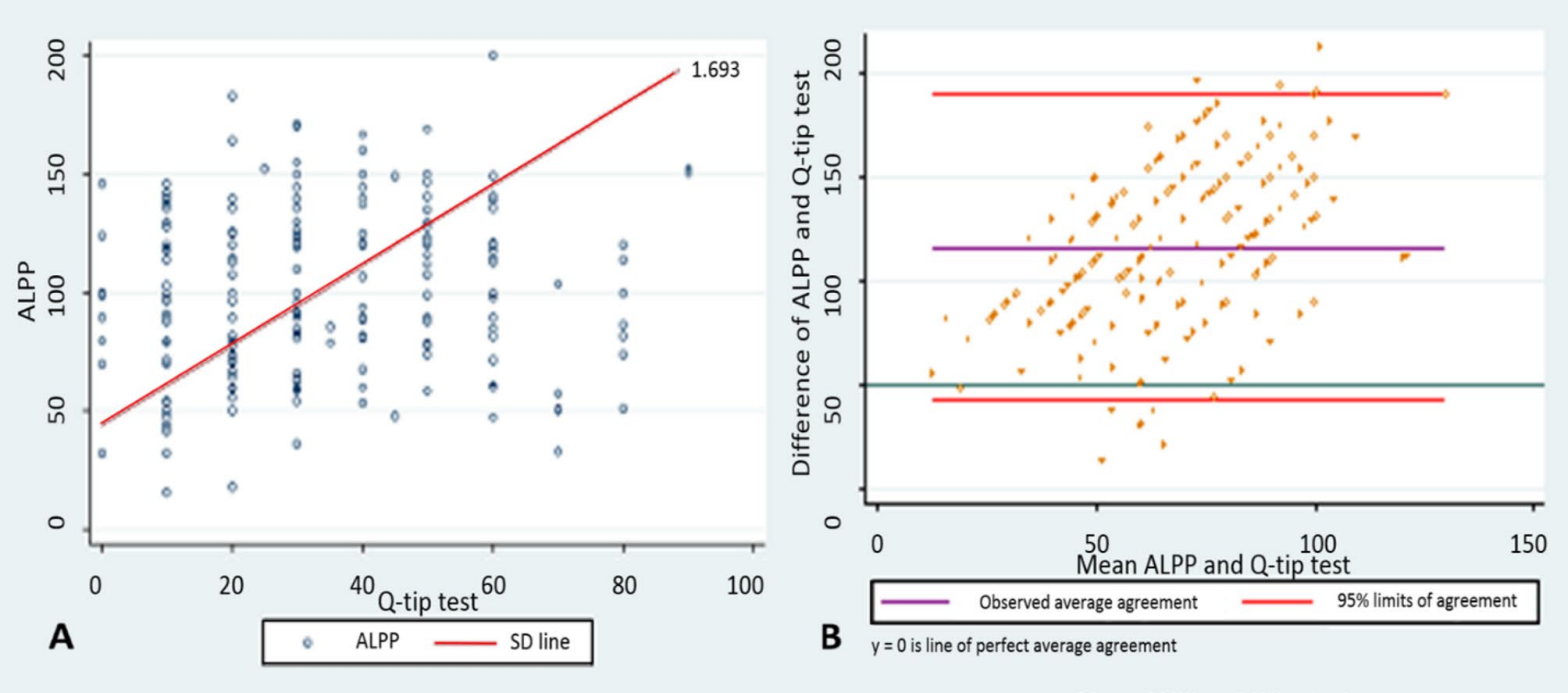
Reduced major axis regression (**A**) and Bland–Altman plots (**B**) of ALPP versus the Q-tip test. In (**A**), a linear relationship was not found when a visual analysis of the two tests was performed. Solid lines in (**B**) indicate mean difference, whereas dashed lines show limits of agreement. Mean (limits of agreement) was 66.6 (− 6.9 to 140.1).

Figure 1 shows the reduced major axis regression and the Bland–Altman plots of ALPP versus the Q-tip test, which shows that no linear relationship was found between these two variables.

## Discussion

As per Blaivas classification, type I SUI is due to hypermobility of the urethrovesical angle, and type II due to rotational descent of the bladder base; the latter being further divided into Type IIa and b, according to the position of the bladder neck relative to the inferior margin of the symphysis pubis^16^. In both the aforementioned, anatomical support mechanisms are lacking (i.e. genuine SUI), contrary to Type III SUI—Intrinsic sphincter deficiency (ISD), which is characterized by a lack of urethral mucosa coaptation^17^.

However, it is in the distinction of these two entities (genuine vs. ISD SUI) that the true clinical value ensues, because it affects treatment approach and results^18^. This distinction may also have greatest clinical relevance in cases of 'fixed' urethra or significantly low ALPP. Several surrogate (and predictive) tools have emerged, notably urethral pressure profilometry, which provides a measurement of the maximum urethral closure pressure (MUCP); and urodynamic abdominal LPP; with values ≤ 20 and ≤ 60 cm H_2_O, respectively, being suggestive of ISD^19–22^. As a drawback, both of these entail invasive testing.

The Q-tip test (or cotton swab test) was introduced in the early 1970s as a straightforward, inexpensive strategy to help diagnose pelvic floor defects in women with SUI. It is useful for identifying urethral hypermobility, which is defined as a deflection angle ≥ 30° from the horizontal during Valsalva. Crystle et al. first described it and used it to distinguish between type I and II SUI; and it was later employed to aid in defining which type of anti-incontinence intervention suited the patient^23^. Since then, studies about proper technique^24^ and reliability of the test^25^ have been published. Considerations about this examination like its reproducibility and its low correlation with urethral mobility measured by ultrasonography or urethrocystography^26,27^. Nowadays a fixed urethra (deflection angle < 30°) is thought to correlate with ISD. Nonetheless, research has been consistently contradictory when assessing the Q-tip test ability to diagnose SUI^28^, discriminating between the SUI types, associating it to urodynamic parameters and even relating it to surgical failure rates^19^.

Promising results are shown by Lemack et al.^29^ in a subanalysis of the SISTEr Trial, a large multicenter randomized study comparing two surgical techniques: the modified Tanagho Burch procedure and the autologous rectus fascial sling procedure for the treatment of SUI. Amongst preoperative assessment, besides UDS and Valsalva LPP measures, several non-invasive tools and parameters where taken into account, including the Q-tip test which was reported at rest, with strain and as a delta. On bivariate analysis authors found a statistically significant association between VLPP (dependent variable), Q-tip straining angle (p < 0.0002) and Q-tip angle delta (p < 0.0046) (independent variables). However, multivariate analysis failed to show this association.

Later, a study by Richter et al.^30^ evaluated predictors for treatment failure following retropubic (TVT) or transobturator (TOT) midurethral sling surgery, based on the results from one of the most relevant SUI studies, the Trial of Mid-Urethral slings (TOMUS). The TOMUS study evaluated 597 women randomized to either treatment and reported follow-up data on 565 patients for up to a year. Failure was defined as objective if the woman presented a positive stress test, a positive 24-h pad test or underwent retreatment; and was reported as subjective based on self-reported UI questionnaires, presence of leakage in a 3-day bladder diary, and/or retreatment. The Q-tip test result (suggesting a fixed urethra) was found to be predictive of failure in both bivariate and multivariate analysis. On bivariate analysis Q-tip delta < 30° showed an OR of 1.95 (95% CI 1.32 – 2.89; p = 0.001) and maximum straining angle < 30^o^, an OR of 1.97 (95% CI 1.23 – 3.14; p = 0.005). On multivariate analysis, the latter showed an OR of 1.89 (95% CI 1.16 – 3.0; p = 0.01). Other statistically significant predictive variables were previous UI surgery, presence of urge UI on validated questionnaires and a 10 g pad test weight^30,31^.

Based on these prior results we aimed to find a correlation between ALPP and Q-tip test results that would aid in predicting ISD, but, although we found that 58.8% of woman with diverse SUI severity had a positive Q-tip test, correlation and concordance between these two variables was poor, even after selecting for severity using high ICIQ-UI scores by creating a composite variable.

Similar to our study, Kreder et al.^28^ implied in a retrospective study evaluating women subjected to sling cystourethropexy and periurethral collagen injection based on whether ISD was present or not. Among ISD patients, 20 (40%) had urethral hypermobility, 7 (35%) of which had a prior history of incontinence surgery. Regarding this issue, the differentiation between the two conditions is recognized as a historical fact but of limited clinical value as it is widely recognized that a certain degree of ISD may be associated with urethral hypermobility leading to an overlap between these two conditions^28,32^.

Another study published in 2003 by Fleischmann et al.^33^ compared the relationship between ALPP, urethral hypermobility and severity of incontinence. This study reports that with ALPP values of < 60, 60–90 and > 90 cm H_2_0, urethral hypermobility was found in 25%, 31% and 41% respectively, without a statistically significant difference between these groups. Additionally, there was no difference in the severity of symptoms. Comparable to our study, the authors concluded that neither a change in urethral mobility nor the severity of symptoms correlate with ALPP. Nevertheless, the potential utility for Q-tip in addition to ALPP test is known for the ability to predict postoperative outcomes. Patients with high urethral mobility have better outcomes in terms of incontinence cure tan those without Q-tip test < 30 degrees even when ALPP indicates a more dysfunctional sphincter^34^.

Strengths of this study include its longitudinal prospective design which reduces recall bias in evaluating UI symptoms and a standardized technique for UDS. Limitations may be provided by the fact that physical examination and the Q-tip were performed by different examiners which may give rise to interobserver differences, however standardization of the technique and compliance to good urodynamic practice guidelines were employed to mitigate this risk.

## Conclusions

Our results show that neither the degree of urethral mobility, nor the composite variables correlate or agree with urethral function tests in UDS, specifically LPP; suggesting ALPP cannot be predicted using the Q-tip test or the ICIQ-SF for classifying patients with ISD-SUI.

Negative results provided by our study should thus be viewed in regard to the bulk of evidence pointing to ISD being a complex entity. ISD diagnosis requires an amount of clinical suspicion, thorough history and physical evaluation, and cumbersome testing, in most cases incorporating invasive urodynamic and videourodynamic evaluation; all of this directed to prevent recurrence, treatment failure and retreatment, which ultimately affects the patient and her quality of life.
